# Applying Pulse Spectrum Analysis to Facilitate the Diagnosis of Coronary Artery Disease

**DOI:** 10.1155/2019/2709486

**Published:** 2019-06-03

**Authors:** Yi-Chia Huang, Yu-Hsin Chang, Shu-Meng Cheng, Sunny Jui-Shan Lin, Chien-Jung Lin, Yi-Chang Su

**Affiliations:** ^1^Graduate Institute of Chinese Medicine, College of Chinese Medicine, China Medical University, Taichung 40402, Taiwan; ^2^Department of Chinese Medicine, Tri-Service General Hospital, National Defense Medical Center, Taipei 11490, Taiwan; ^3^ChanDer Clinic, Taichung 40356, Taiwan; ^4^Department of Internal Medicine, Tri-Service General Hospital, National Defense Medical Center, Taipei 11490, Taiwan; ^5^ChanDer Clinic, Taipei 10646, Taiwan; ^6^School of Chinese Medicine, College of Chinese Medicine, China Medical University, Taichung 40402, Taiwan; ^7^National Research Institute of Chinese Medicine, Ministry of Health and Welfare, Taipei 11221, Taiwan

## Abstract

Not all patients with angina pectoris have coronary artery stenosis. To facilitate the diagnosis of coronary artery disease (CAD), we sought to identify predictive factors of pulse spectrum analysis, which was developed by Wang and is one technique of modern pulse diagnosis. The patients suffered from chest pain and received cardiac catheterization to confirm the CAD diagnosis and Gensini score were recruited. Their pulse waves of radial artery were recorded. Then, by performing a fast Fourier transform, 10 amplitude values of frequency spectrum harmonics were obtained. Each harmonic amplitude was divided by the sum of all harmonic amplitude values, obtaining the relative percentages of 10 harmonics (C1-C10). Subsequently, multivariate logistic regression was conducted with two models and the areas under the receiver operating characteristic curves (ROC) of these 2 models were compared to see if combining the pulse diagnosis parameters with the risk factor of CAD can increase the prediction rate of CAD diagnosis. The predictive factors of CAD severity were analyzed by multivariate linear regression. A total of 83 participants were included; 63 were diagnosed CAD and 20 without CAD. In the CAD group, C1 was greater and C5 was lower than those of the non-CAD group. The CAD risk factors were put alone in Model 1 to perform the multivariate logistic regression analysis which had a prediction rate of 77.1%; while putting the C1 and C5 harmonics together with the risk factors into Model 2, the prediction rate increased to 80.7%. Finally, the area under ROC of Model 1 and Model 2 was 0.788 and 0.856, respectively. Furthermore, left C1, left C5, gender, and presence of hyperlipidemia were predictors of CAD severity. Therefore, pulse spectrum analysis may be a tool to facilitate CAD diagnosis before receiving cardiac catheterization. The harmonics C1 and C5 were favorable predictive indicators.

## 1. Introduction

Coronary artery disease (CAD) is a common cardiovascular disease. In recent years, the prevalence and mortality of CAD have been high [1] and are predicted to become a severe economic burden by 2020 [2]. Risk factors affecting CAD include gender, age, obesity, smoking, exercise, hypertension, diabetes, and high blood cholesterol [3]. At present, clinical evaluations of CAD can be conducted through electrocardiograms, nuclear medicine scans, tomography, or cardiac catheterization. However, according to the clinical guidelines of the American Heart Association, only cardiac catheterization can confirm diagnosis of CAD [4].

CAD is caused by atherosclerosis of the coronary arteries. Such arteries result in insufficient blood flow and oxygen supply and can cause myocardial ischemic changes which lead to a typical clinical manifestation of CAD: angina pectoris or chest pain. However, not all patients with angina pectoris or chest pain have CAD. A previous study indicated that 60% of patients who underwent cardiac catheterization for suspected CAD due to chest pain or angina pectoris did not have narrowed coronary arteries [5]. In addition, patients may be unwilling to receive further examination because of the invasiveness of the cardiac catheterization. But it is fatal if this is CAD indeed. The rupture of the plaque will cause death due to acute myocardial infarction. The probability of having CAD can be estimated by the risk factors. Therefore, if there are noninvasive tools which can provide better reference for clinicians, especially noncardiologists, so that the clinicians can refer patients who has higher probability having CAD to receive cardiac catheterization. Thus, assisted by the noninvasive tools, the risk of myocardial infarction may be reduced.

The method “pulse spectrum analysis” was proposed by Wang based on the “resonance of organs with the heart” theory to explain the correlation between the pulse and human organs [6–8]. The “pulse” is a composite wave composed of harmonics with different frequencies and can be transformed into resonant harmonics of different frequencies by Fourier analysis. Wang postulated that the pulse was jointly formed from the unidirectional flow produced by heart contractions and the pulsatile flow produced by the arteries during diastole. Because the arterial networks in organ tissue throughout the body have different resistances, resonance is produced to form harmonics with different frequencies. This results in differences in the blood flow entering the tissues of each organ [9]. Organ tissues that receive the same frequency of harmonics and obtain blood flow can thus be categorized as a group. Therefore, observations of changes in these harmonics can be used to infer the conditions of organs. This theory was verified through observations of pulse differences between patients with different diseases and those without them, as well as observations of pulse changes after acupuncture [10–12] and pharmacological interventions [13–17].

The application of pulse spectrum analysis to assist the diagnosis of CAD has not been studied yet. This study aims to identify the characteristics of harmonics changes that aid CAD diagnosis in order to provide an evidence for further clinical diagnosis and preventive treatment.

## 2. Methods

### 2.1. Participants

This prospective observational study was conducted from July 2011 to October 2012 in the cardiology ward of a medical center (Tri-Service General Hospital) in Northern Taiwan. Participant inclusion criteria for this study were (1) individuals who were 18 years of age or older, any gender, seeking treatment for chest pain or angina pectoris and (2) outpatients whose conditions were diagnosed by cardiologists as needing to be hospitalized to receive cardiac catheterization in order to confirm diagnosis and treatment. Exclusion criteria included acute myocardial infarction, cardiac arrhythmia, valvular heart disease, cancer, severe infection, pregnancy, severe mental illness, and individuals whose pulses could not be measured.

### 2.2. Diagnosis and Severity of CAD

The diagnosis and severity of CAD was evaluated by cardiologists according to photographic images of blood vessels taken during cardiac catheterization. If image results indicate that ≥ 1 blood vessel was ≥ 50% narrowed, CAD diagnosis was confirmed [18]. Severity is determined by utilizing the Gensini scoring system, a quantification method proposed by Gensini in 1983. Scores are calculated by summing the severity scores of vessel constriction in all areas with coronary artery stenosis × the stenosis grade of the areas. If there is no stenosis in any area, the score is 0 [19].

### 2.3. Instruments

This study adopted the PDS-2010 Skylark Pulse Analysis System (Ministry of Health and Welfare Medical Device License No. 003627; Skylark Device & Systems Co., Ltd., Taiwan) to conduct pulse measurement. This single-probe measurement instrument was equipped with a high-fidelity pressure sensor, as well as X-Y-Z triaxial portable tracks used to locate the probe and apply vertical downward pressure in the measurement area to obtain the analog signal. Then, built-in programs were used to convert the analog signal into digital data and stored it in a computer.

### 2.4. Procedures and Data Collection

Participants were hospitalized from 15:00 to 17:00 the day before they received cardiac catheterization. After history-taking and related examinations were done, researchers explain the study procedure and consent form in detail to participants. Once participants understood and provided signed consent, researchers recorded their age, gender, height, weight, and comorbid diseases (including diabetes, hypertension, and hyperlipidemia). Researchers then guided participants to an independent, quiet examination room with a room temperature maintained at 25–26°C.

**Figure 1:**
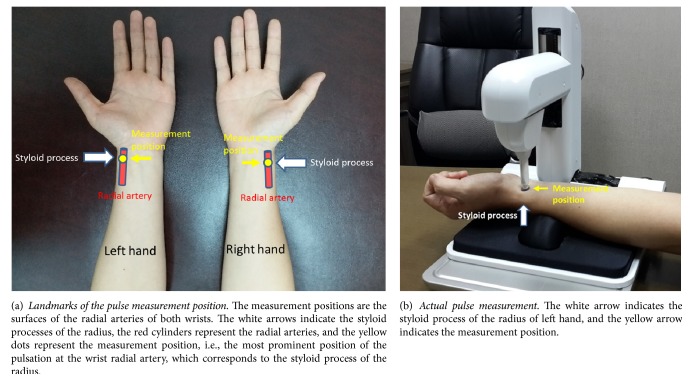


Measurements were performed by researcher using pulse diagnostic instruments after participants had rested quietly in the examination room for 10 minutes. Pulse measurements were performed on the radial arteries of the wrists on both arms, with the right arm followed by the left. In this study, the measurement position we selected was the most prominent position of the pulsation at the wrist radial artery, which corresponded to the styloid process of the radius. The graphical demonstration of the measurement position of the pulse is shown in Figures 1(a) and 1(b). Prior to measuring, we first located the most distinct section of radial artery pulsation and marked it, then positioned the pressure probe on the marking, slowly pressing down until the pulse had emerged to ensure there were no errors in positioning. We then continued to press the pressure probe vertically downward until the pulse with the highest amplitude appeared before recording the measurement. Using the recording mode of the PDS-2010 Skylark Pulse Analysis System, each arm was continuously recorded for 6 s. The analog signals were converted into digital data and stored for further pulse spectrum analysis.

On the following day, cardiac catheterization was performed on participants at the radial artery (main section) or femoral artery. This was followed by coronary angiography to confirm the diagnosis of CAD and calculation of Gensini score. The coronary angiography was performed by the cardiologists, who were blind to the result of pulse measurements.

Additionally, the following information was included recorded from medical chart: blood pressure values at the time of admission; and data such as fasting plasma glucose (FPG), total cholesterol, triglyceride (TG), and low density lipoprotein (LDL) cholesterol from laboratory examinations was arranged by cardiologists based on clinical diagnostic needs.

### 2.5. Pulse Spectrum Analysis

In accordance with the method of pulse spectrum analysis developed by Wang, after we converted the digital data of the pulse measurements into a.txt file, we used LabVIEW 7 (National Instruments Co., USA) to perform a fast Fourier transform and obtained 10 amplitude values of frequency spectrum harmonics (An, n = 1–10). Each harmonic amplitude was then divided by the sum of all harmonic amplitude values (A0), obtaining the relative percentages of the harmonics (Cn, n = 1–10) or Cn = 100%  × (An/A0).

### 2.6. Statistical Analysis

Regarding demographic information for continuous variable, data are expressed as mean±SD and for categorical variables as numbers with corresponding percentages. The Chi-square test was used for categorical variables. Comparisons between CAD and non-CAD groups were performed via Mann–Whitney U test.

In the analysis of pulse diagnosis parameters, to evaluate whether intragroup differences existed, we first conducted the Wilcoxon test on the relative percentages of the harmonics of both arms of the patients in each group. Then, the Mann–Whitney U test was used to evaluate the relative percentages differences of the harmonics between the CAD and non-CAD groups. Subsequently, multivariate logistic regression was conducted with two models: (Model 1) including only CAD risk factors, e.g., age, gender, body mass index (BMI), history of hypertension, diabetes, and hyperlipidemia; (Model 2) including the above CAD risk factors with the relative percentages of the harmonics with significant differences to assess the joint predictive effect on CAD diagnosis. Multivariate linear regression was also performed with the same variables along with the Gensini score to analyze which of these factors affected the severity of CAD. Finally, areas under the receiver operating characteristic curves (ROC) of Model 1 and Model 2 were compared to see if combining the pulse diagnosis parameters can increase the prediction rate of CAD diagnosis.

Statistical calculations were performed using software package SPSS 20.0 (SPSS Inc., Chicago, IL). All comparisons were two-tailed;* P *< 0.05 was regarded as statistically significant.

## 3. Results

### 3.1. Participants

A total of 152 patients met the inclusion criteria. After excluding patients who rejected receiving and were unable to complete the pulse measurement, a total of 83 participants were included. Following cardiac catheterization, there were 63 individuals with CAD and 20 without CAD, and most of them were men. The average age of the CAD group was 59.77 ± 10.51 years, significantly higher than the 50.68 ± 12.64 years of the non-CAD group (*P *< 0.01). There were no significant differences between the two groups in terms of gender, BMI, blood pressure values, the proportions with comorbid diseases of hypertension, diabetes, and hyperlipidemia (Table 1).

Not all participants received the same biochemistry examinations upon admission due to differences in evaluation among clinical physicians. The number of people who received the examination is indicated in parentheses. There were no significant differences in the values of FPG, total cholesterol, LDL cholesterol, or TG between the CAD and non-CAD group (Table 1).

### 3.2. Prediction of CAD Diagnosis and Severity Using Pulse Spectrum Analysis

First, the comparison of the relative percentages of the harmonics measured in the radial artery pulse of left and right arm of the CAD or non-CAD group was examined, respectively, by Wilcoxon signed-rank test. The results revealed that regardless of whether participants were diagnosed with CAD, there was no difference in the relative percentages of the harmonics between the left and right radial artery pulse (Table 2).

Subsequently, the comparison of the CAD and non-CAD groups on the respective relative percentages of the harmonics of the left or right radial artery pulse was examined, respectively, by the Mann–Whitney *U* test. The results showed significant differences between C1 and C5 in both arms. In the CAD group, C1 was greater and C5 was lower than those of the non-CAD group (Table 3).

Then, the CAD risk factors were put alone or together with the respective relative percentages of the harmonics (C1 and C5, both left and right artery pulse) to perform the multivariate logistic regression analysis using an enter model, respectively, to realize the joint predictive of C1 and C5 on CAD diagnosis (Tables 4 and 5). Among the CAD risk factors, age (RR = 1.07, 95% CI: 1.01–1.13,* P *< 0.05) had statistical significance in Model 1, which had a prediction rate of 77.1% (Table 4). While putting the respective relative percentages of the harmonics (C1 and C5, both left and right artery pulse) into the model, the prediction rate increased to 80.7%. The statistically significant factors in Model 2 were gender (RR = 4.80, 95% CI: 1.03–22.40,* P *< 0.05) and left C5 (RR = 0.74, 95% CI: 0.53-0.96,* P *< 0.05).

In order to explore the predictive factors of CAD severity, a multivariate linear regression analysis was performed with the Gensini score and the same factors (C1, C5, age, gender, BMI, presence of hypertension, presence of diabetes, and presence of hyperlipidemia) by an enter manner. The results revealed that the left C1, left C5, gender, and presence of hyperlipidemia were predictors of CAD severity (Table 6). Therefore, the increase in left C1 and decrease in left C5 are both predictive factors for CAD diagnosis and the Gensini score.

**Figure 2:**
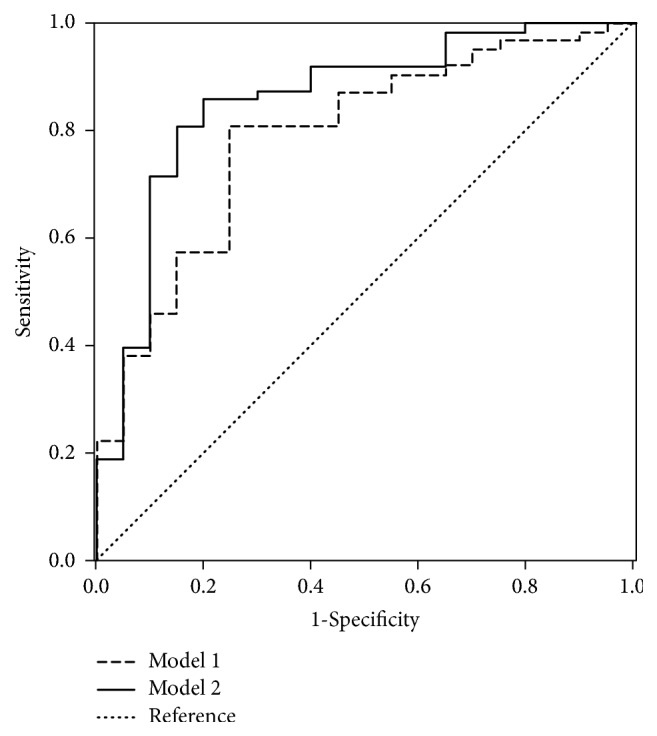
*Receiver operator curves (ROC) comparing Model 1 (influence factors: age, gender, BMI, hypertension, diabetes, and hyperlipidemia) with Model 2 (influence factors and relative spectral energy values) for predicting coronary artery disease.* Area under curve (AUC) of Model 1 and Model 2 is 0.788 and 0.856, respectively.

Finally, the area under ROC of Model 1 and Model 2 was 0.788 and 0.856, respectively (Figure 2).

## 4. Discussion

To the best of our knowledge, this was the first study to explore if pulse spectrum analysis can increase the prediction rate of CAD diagnosis in patients with chest pain or angina pectoris prior to cardiac catheterization. According to our findings, applying the pulse spectrum analysis together can receive a higher prediction rate for CAD diagnosis; specifically, the relative percentages of harmonics C1 and C5 are favorable predictive indicators. In patients diagnosed with CAD, there was a clear correlation when an increase in C1 and a decrease in C5 were observed.

The pulse spectrum analysis method used in the present study was developed by Wang in accordance with organ resonance theory [6, 20]. Wang proposed that the pulse signals measured from the radial artery in the wrist are composed of several harmonics. The harmonic amplitudes converted from these pulse waves become increasingly smaller; those after the 10th harmonic can be ignored because they are excessively small. The relative percentages of the harmonics produced from the normalization of each harmonic amplitude can be used to reflect the condition of organs. In accordance with experimental observations and Chinese medicine theory, Wang proposed that the corresponding relationships were as follows: C1 corresponds to the liver, C2 to the kidneys, C3 to the spleen, C4 to the lungs, C5 to the stomach, C6 to the gallbladder, C7 to the bladder, C8 to the large intestine, C9 to the triple energizers, and C10 to the small intestine. In patients with different diseases, changes will appear in different corresponding areas [15, 21, 22]. Several previous studies have used this analysis method to investigate correlations between diseases. These include observations of death and prognosis in patients with terminal cancer [23]; changes during fasting [24]; and comparisons of differences between patients with mania [25], child patients with atopic eczema [26], and patients without these conditions. In these studies, distinct differences can be observed in the related spectrum.

CAD is a heart disease that progresses from pathological changes in the coronary arteries. However, unless it involves severe heart damage owing to acute myocardial infarction, regular CAD develops slowly. In the development of CAD, changes in the circulation of other organs are interconnected or compensated and then redistributed. Therefore, we excluded patients with acute myocardial infarction from our observations and hoped that outpatients would demonstrate the aforementioned phenomenon; our results also indicated points of difference. When participants were diagnosed with CAD, C1 and C5 demonstrated significant changes, which indicated that the expected response had occurred in these two organ groups.

Previous studies have been conducted using pulse spectrum analysis to observe patients with CAD; however, these studies have been scarce and had contrasting findings. In 1993, Chen observed pulse changes in 17 patients with acute myocardial infarction and measured their relative percentages of harmonics on the day that acute myocardial infarction occurred, 1 day after occurrence, 2 days after occurrence, and after creatine kinase (CK) had returned to a normal value. The results demonstrated that although C2 and C3 decreased during occurrence and increased in the following days, the other harmonics did not demonstrate differences before and after occurrence [27]. This study conducted research on patients with acute myocardial infarction without using a control group. Therefore, this type of change may have been an acute response that did not produce a compensatory regulatory response in the circulation of organs and thus differed from our findings. Additionally, in 2008, Kuo compared pulse diagnosis in 22 patients prepared to receive cardiac catheterization due to CAD diagnosis with a control group of 13 regular patients [28]. The results found that only the C3 of the left arm reached a statistically significant difference; namely, the C3 of patients with CAD was greater than that of patients without CAD. The present study also found similar differences in this area, but they did not attain statistical significance. The C1 value was greater in the patients without CAD, whereas C5 was greater in the left arms of patients with CAD. The right arm, by contrast, was similar to our results and greater in the patients without CAD. However, these results did not achieve statistical significance. In this study, the cardiac catheterization results of the patients in the CAD group were not specified, their diagnosis and severity were not differentiated, the conditions of the regular healthy group were not explained, and the sample size was small. This may have produced the differences with the results of our study.

Our result showed that C1 of the radial pulse wave could be a favorable predictive indicator for CAD diagnosis. The gradual increasing of C1 by aging was investigated by the harmonic based pulse waveform analysis method [29]. Other studies also reported the factor of atherosclerosis [30, 31], or increasing afterload [29] will increase the first harmonic impedance which is related to the increasing burden of heart muscle. Chang et al. reported that screening for the C1 may improve the risk stratification of cardiac events and silent coronary artery disease in asymptomatic patients [32]. Hence, the first harmonic amplitude increment could be a noninvasive risk marker for cardiovascular disease. However, further studies should be investigated for confirm interaction between the harmonic based radial pulse analysis and coronary artery disease.

Our result showed that the C5 in the CAD group was lower than those of the non-CAD group; we proposed that the cerebral blood flow of the CAD group might be lower than the non-CAD group. According to the previous research, several studies have found evidence of a high prevalence of abnormalities in cerebral blood flow in patients with cardiac small-vessel disease [33]. Pai et al. reported that in patients with cardiac small-vessel disease, brain hypoperfusion lesions on technetium-99m ethyl cysteinate dimer brain SPECT were common and positively associated with the presence and extent of abnormalities in myocardial perfusion as revealed by thallium-201 myocardial perfusion SPECT [34]. Sun et al. found that 23 out of the 25 cases with definite myocardial perfusion defects diagnosed by thallium-201 myocardial perfusion SPECT also had multiple hypoperfusion areas in the brain [35]. Hsiu et al. found that there was a significant positive correlation between C5 and cerebral blood flow using laser Doppler flowmetry during acupuncture intervention in stroke patient [36]. The correlation among measurements of cerebral blood flow using ultrasound [37], phase-contrast magnetic resonance imaging [38], brain perfusion SPECT [39], or 99mTc-ECD [40] could be further explored and validated.

The left C1 and C5 change is more prominent compared to the right in our study. In human anatomy, the left subclavian artery and left common artery come directly off the aortic arch on the left side of the body, while on the right side they arise from the relatively short brachiocephalic artery when it bifurcates into the right subclavian artery and the right common carotid artery. Hence, the changes of left C1 and C5 are more prominent compared to the right side which might be related to the fact that the vibration resistance is smaller in the left side which is directly off the aortic arch than in the right side which is passing the bifurcation. According to the previous studies, the major wave modes in large arteries are the variation of the cross-sectional area of the artery driven by the pulsatile pressure force [41]. Hence, the pressure-area wave equation [42] plays the role of the master equation for pulse waves. The periodic changing of the cross-sectional area in the arterial tube is a transverse wave with large longitudinal tension [43]. In this mechanism, the pulse wave velocity is related to the longitudinal elasticity property of the artery; and the wall is the major medium for the wave propagation [42]. Wang et al. assumed that the primary power consumption in delivering the blood from the left ventricle to the veins is only the dissipation in flow viscosity and in vibration resistance after the ventricular-arterial system reaching a steady state and the whole arterial system will execute distributed stationary vibrations [44]. However, more studies are suggested to confirm the hypothesis.

The present study had some limitations. First, the sample size was small and the study was conducted in one medical center in Northern Taiwan. The participant population was simple, which may have caused sample selection bias. Therefore, if the findings of this study are to be generalized to all patients with CAD, a large-sample, multiregional, and multiunit study must be conducted for verification.

Second, although the presence of CAD causes myocardial ischemia or necrosis, which leads to angina pectoris or death, patients without CAD may also have heart damage. Because no nuclear medicine cardiac scan record can be used to evaluate the degree of pathological changes in the heart, the correlation between pulse spectrum analysis results and these changes could not be directly compared.

Third, in accordance with research ethics, invasive cardiac catheterization should not be performed on individuals without CAD. Therefore, the present study did not determine that asymptomatic and non-CAD patients could be compared. The findings are only suitable for the evaluation of symptomatic, suspected CAD patients.

## 5. Conclusions

We explored the pulse spectrum analysis to determine that the relative percentages of C1 and C5 harmonics were favorable indicators which can increase the prediction rate of CAD diagnosis. This method can facilitate the CAD diagnosis before cardiac catheterization is undertaken to prevent the risk of myocardial infarction.

## Figures and Tables

**Table 1:** Demographic features of patients with and without coronary artery disease.

Variable	CAD	Non-CAD	*P* value
	(n=63)	(n=20)	
Gender (male: n, %)	52(82.5%)	14(70.0%)	NS
Age (yr)	59.77±10.51	50.68±12.64	<0.01
Height (cm)	166.31±8.09	167.40±7.73	NS
Weight (kg)	71.45±12.04	76.75±15.62	NS
BMI (kg/m^2^)	25.81±3.75	27.23±4.17	NS
Comorbidity			
Hypertension (n, %)	42(66.7%)	10(50.0%)	NS
Diabetes (n, %)	17(27.0%)	2(10.0%)	NS
Hyperlipidemia (n, %)	23(36.5%)	7(35.0%)	NS
SBP (mmHg)	135.19±15.10	135.60±21.77	NS
DBP (mmHg)	80.89±9.91	82.25±10.03	NS
FPG (mg/dl)	153.14±60.53(57)	124.88±35.96(17)	NS
Total cholesterol (mg/dl)	171.45±43.16(56)	155.94±22.64(16)	NS
TG (mg/dl)	142.31±65.21(61)	127.61±66.66(18)	NS
LDL (mg/dl)	115.96±42.56(57)	101.69±24.99(16)	NS

(i) Continuous data are presented as mean ± SD.

(ii) Categorical data are presented as number of patients (percentages).

(iii) For FPG, total cholesterol, LDL, and TG, the number of people examined is indicated in parentheses.

(iv) *P* < 0.05: being statistically significant.

(v) NS: not significant.

(vi) BMI: body mass index = Weight (kg)/ Height^2^ (m)

(vii) CAD: coronary artery disease; SBP: systolic blood pressure; DBP: diastolic blood pressure; FPG: fasting plasma glucose; TG: triglyceride; and LDL: low density lipoprotein.

**Table 2:** Comparison of relative percentages of the harmonics in the radial artery pulse of both arms of patients with and without CAD.

	CAD(n=63)			Non-CAD(n=20)		
	Left	Right	*P* value	Left	Right	*P* value
C1	52.04±6.93	50.38±7.24	NS	48.03±6.04	46.33±4.86	NS
C2	28.56±5.91	27.78±5.54	NS	28.42±7.29	26.61±4.21	NS
C3	17.34±5.33	17.06±4.68	NS	17.10±5.50	16.44±3.38	NS
C4	8.78±3.96	8.58±3.60	NS	8.78±3.94	8.62±2.84	NS
C5	6.12±2.53	6.10±2.11	NS	8.02±3.24	7.70±2.22	NS
C6	5.17±1.94	5.57±2.06	NS	6.40±2.65	6.49±2.48	NS
C7	3.65±2.03	3.95±1.95	NS	3.93±1.98	4.20±2.08	NS
C8	2.43±1.48	2.58±1.47	NS	2.56±1.28	2.74±1.25	NS
C9	1.73±1.13	1.92±1.01	NS	2.12±1.12	2.28±0.84	NS
C10	1.37±0.73	1.54±0.82	NS	1.60±0.97	1.83±0.77	NS

(i) Continuous data are presented as mean ± SD.

(ii) Values have been calculated using Wilcoxon signed-rank test.

(iii) P < 0.05: being statistically significant.

(iv) NS: not significant.

**Table 3:** Comparison of relative percentages of the harmonics in the radial artery pulse in patients with and without CAD.

	Left radial artery pulse			Right radial artery pulse		
	CAD (%)	Non-CAD (%)	*P* value	CAD (%)	Non-CAD (%)	*P* value
C1	52.04±6.93	48.03±6.04	<0.01	50.38±7.24	46.33±4.86	0.01
C2	28.56±5.91	28.42±7.29	NS	27.78±5.54	26.61±4.21	NS
C3	17.34±5.33	17.10±5.50	NS	17.06±4.68	16.44±3.38	NS
C4	8.78±3.96	8.78±3.94	NS	8.58±3.60	8.62±2.84	NS
C5	6.12±2.53	8.02±3.24	<0.01	6.10±2.11	7.70±2.22	0.02
C6	5.17±1.94	6.40±2.65	NS	5.57±2.06	6.49±2.48	NS
C7	3.65±2.03	3.93±1.98	NS	3.95±1.95	4.20±2.08	NS
C8	2.43±1.48	2.56±1.28	NS	2.58±1.47	2.74±1.25	NS
C9	1.73±1.13	2.12±1.12	NS	1.92±1.01	2.28±0.84	NS
C10	1.37±0.73	1.60±0.97	NS	1.54±0.82	1.83±0.77	NS

(i) Continuous data are presented as mean ± SD.

(ii) Values have been calculated using Mann–Whitney *U* test.

(iii) P < 0.05: being statistically significant.

(iv) NS: not significant.

**Table 4:** Multivariate analysis of influence factors for coronary artery disease.

Prediction rate: 77.1%					
Variable	B	Wald	Relative risk	95% CI	*P* value
Age	0.07	6.14	1.07	1.01-1.13	<0.05
Gender	1.37	3.84	3.94	1.00-15.52	0.05
BMI	-0.10	1.50	0.91	0.78-1.06	NS
Hypertension	0.57	0.81	1.77	0.51-6.16	NS
Diabetes	1.06	1.44	2.89	0.51-16.37	NS
Hyperlipidemia	0.25	0.15	1.28	0.37-4.49	NS

(i) Multivariate logistic regression analysis was performed by “enter” method.

(ii) P < 0.05: being statistically significant.

(iii) NS: not significant.

(iv) CI: confidence interval.

**Table 5:** Multivariate analysis of relative spectral energy values and influence factors for coronary artery disease.

Prediction rate: 79.5%					
Variable	B	Wald	Relative risk	95% CI	*P* value
Age	-0.002	0.004	1.00	0.93-1.07	NS
Gender	1.57	3.99	4.80	1.03-22.40	<0.05
BMI	-0.06	0.51	0.94	0.80-1.11	NS
Hypertension	0.61	0.66	1.84	0.42-7.99	NS
Diabetes	1.59	1.93	4.93	0.52-46.59	NS
Hyperlipidemia	0.21	0.08	1.23	0.30-5.10	NS
Right C1	0.10	1.88	1.11	0.96-1.28	NS
Right C5	-0.22	1.55	0.80	0.57-1.14	NS
Left C1	0.09	1.17	1.09	0.93-1.28	NS
Left C5	-0.34	5.12	0.71	0.53-0.96	<0.05

(i) Multivariate logistic regression analysis was performed by “enter” method.

(ii) P <0.05: being statistically significant.

(iii) NS: not significant.

(iv) CI: confidence interval.

**Table 6:** Linear regression analysis of relative spectral energy values and influence factors for the severity of coronary artery disease.

Variable	B	t	*P* value
Age	-0.28	-0.80	NS
Gender	24.26	2.96	<0.01
BMI	-0.35	-0.39	NS
Hypertension	0.04	0.01	NS
Diabetes	14.58	1.90	NS
Hyperlipidemia	15.14	2.26	0.03
Right C1	0.84	1.45	NS
Right C5	0.16	0.10	NS
Left C1	1.76	3.00	<0.01
Left C5	-3.98	-3.05	<0.01

(i) Multivariate linear regression analysis was performed by “enter” method.

(ii) The severity was

(iii) P <0.05: being statistically significant.

(iv) NS: not significant.

## Data Availability

The data derived from experiments on human subjects to support the findings of this study are available from the corresponding author upon request.
